# Complete genome sequence of *Aquabacterium* sp. strain TM01, a membrane-adherent bacterium isolated from a membrane bioreactor treating municipal sewage

**DOI:** 10.1128/mra.00476-26

**Published:** 2026-05-29

**Authors:** Toru Miwa, Tomoki Iwashita, Masashi Hatamoto, Hideyuki Tamaki

**Affiliations:** 1 Biomanufacturing Process Research Center, National Institute of Advanced and Industrial Science and Technology (AIST) 73459, Tsukuba, Ibaraki, Japan; 2 Department of Civil and Environmental Engineering, Nagaoka University of Technology 52756, Niigata, Japan; Queens College Department of Biology, Queens, New York, USA

**Keywords:** MBR, biofilm, EPS, biofouling

## Abstract

We report the complete genome sequence of *Aquabacterium* sp. strain TM01, a membrane-adherent bacterium isolated from activated sludge in a labooratory-scale membrane bioreactor treating municipal sewage. The complete genome is 3,785,964 bp with a G + C content of 64.4%.

## ANNOUNCEMENT

The genus *Aquabacterium* was first isolated from biofilms in Berlin drinking water distribution system (1). The genus currently includes 12 species found in various environments and is characterized by the ability to degrade complex organic compounds and adapt to low-nutrient environments (1–6). Despite its known dominance in membrane bioreactor (MBR) systems (7, 8), no validly published strain of *Aquabacterium* has been isolated from an MBR to date. Here, we report the complete genome sequence of *Aquabacterium* sp. strain TM01, isolated from a laboratory-scale MBR treating municipal sewage.

Activated sludge collected from the lab-scale MBR at sewage treatment plant (37.48°N, 138.85°E) on 25 May 2025 was used as seed sludge (9, 10). Activated sludge supernatant (ASSP) medium was prepared by centrifugation and autoclaved. A piece of membrane was submerged into the ASSP medium inoculated with the seed sludge and incubated at 30°C aerobically. Every week, the membrane was washed with Milli-Q water, and the ASSP was replaced with fresh ASSP. After three cycles, the membrane was transferred to PBS, vortexed to detach attached cells, and the resulting suspension was dropped onto ASSP agar plates. A single colony, designated TM01, was isolated from the plate after incubation at 30°C for 1 week. The isolate was cultivated in R2A broth at 30°C for 4 days. Genomic DNA was extracted from centrifuged cells using the phenol-chloroform method (11). The genomic DNA was mechanically sheared to a target fragment size of approximately 10–25 kb using a Megaruptor 3 (Diagenode). A PCR-free SMRTbell library was prepared using the SMRTbell Prep Kit 3.0 (PacBio). Polymerase binding was performed using the Revio SPRQ polymerase kit (PacBio), and sequencing was performed on a PacBio Revio. HiFi reads were generated from raw subreads using SMRT Link (v25.2.0.266456) by constructing circular consensus sequences (CCSs) and removing reads with a mean quality value below Q20. A total of 17,799 HiFi reads were obtained (read N_50_ = 16,078 bp; mean Q30 = 90.2%). Reads shorter than 1,000 bp and those in the lowest 10% of read quality scores were removed using Filtlong (v0.2.1). Genome assembly was performed using Flye v2.9.3-b1797 (12), with three polishing iterations. Genome annotation was performed using Prokka v1.14.6 (13). Genome completeness and contamination were assessed using CheckM2 v1.0.1 (14). All tools were run with default parameters, unless otherwise specified.

The complete genome of strain TM01 consists of two contigs totaling 3,785,964 bp (G + C content, 64.4%). The genome encodes 3,378 CDSs, 12 rRNA genes (4 complete 16S-23S-5S operons), and 64 tRNA genes (Table 1). Similarity of the full-length 16S rRNA gene from the genome assembly was 98.2% against the closest type of strain *Aquabacterium commune* DSM 11901. The whole-genome average nucleotide identity value calculated using the OrthoANIu algorithm (15) was 79.8% against *A. commune* DSM 11901, well below the species boundary threshold, suggesting that strain TM01 represents a novel species. These genomic data provide a valuable resource for understanding the ecological roles and metabolic potential of *Aquabacterium* species in membrane bioreactor systems.

**TABLE 1:** Overview of the complete genome sequence of the strain TM01

Parameter	Value for TM01
Genome size (bp)	3,785,964
No. of contigs	2
G + C content (%)	64.4
Sequencing coverage (×)	65.4
No. of raw reads	17,799
Read N50 (bp)	16,078
Mean read length (bp)	13,920
No. of CDSs	3,378
No. of rRNA genes	12 (4 operons)
No. of tRNA genes	64
CheckM2 Completeness (%)	100
CheckM2 Contamination (%)	0.17

## Data Availability

The complete genome sequence of *Aquabacterium* sp. strain TM01 has been deposited in DDBJ/ENA/GenBank under accession numbers AP046528 and AP046529. The raw sequencing reads have been deposited in the DDBJ Sequence Read Archive under the accession number DRR916325. The BioProject accession number is PRJDB40516, and the BioSample accession number is SAMD01828536.
